# Transient Ischemic Attack Secondary to Device-Related Thrombus After Left Atrial Appendage Closure

**DOI:** 10.7759/cureus.24073

**Published:** 2022-04-12

**Authors:** Khandakar M Hussain, Ashish Jain, Nuzhat T Ahmad, K.M. Anwar Hussain, Salah Aldergash

**Affiliations:** 1 Internal Medicine, Conemaugh Memorial Medical Center, Johnstown, USA; 2 Internal Medicine, Sylhet MAG Osmani Medical College, Johnstown, USA; 3 Cardiology, Conemaugh Memorial Center, Johnstown, USA

**Keywords:** systemic thromboembolism, stroke, watchman device, left atrial appendage closure, device related thrombus

## Abstract

The patient is an 84-year-old female with a significant past medical history of traumatic subarachnoid hemorrhage, bleeding peptic ulcer disease, permanent atrial fibrillation status post percutaneous left atrial appendage closure (LAAC) initially admitted to the hospital secondary to expressive aphasia. The patient was found to have a transient ischemic attack (TIA). A transesophageal echocardiogram (TEE) showed a thrombus on the watchman device (WD). The patient was treated with unfractionated heparin infusion and later transitioned to apixaban without any further TIA or stroke over 30 days period. Device-related thrombosis (DRT) with systemic thromboembolism occurred almost after 480 days of putting the WD which is very rare.

## Introduction

For patients with nonvalvular atrial fibrillation and contraindication for systemic anticoagulation, percutaneous left atrial appendage closure (LAAC) surgery is an established strategy [1-3]. Recent multiple studies, however, highlighted the clinical impact of device-related thrombosis (DRT) and systemic thromboembolism [4-5]. The most common systemic thromboembolism occurs within six months of the device implantation. We present a case where systemic thromboembolism from device thrombosis occurred after 480 days of the initial procedure and normal post-procedure transesophageal echocardiogram (TEE) at day 45.

## Case presentation

**Figure 1:**
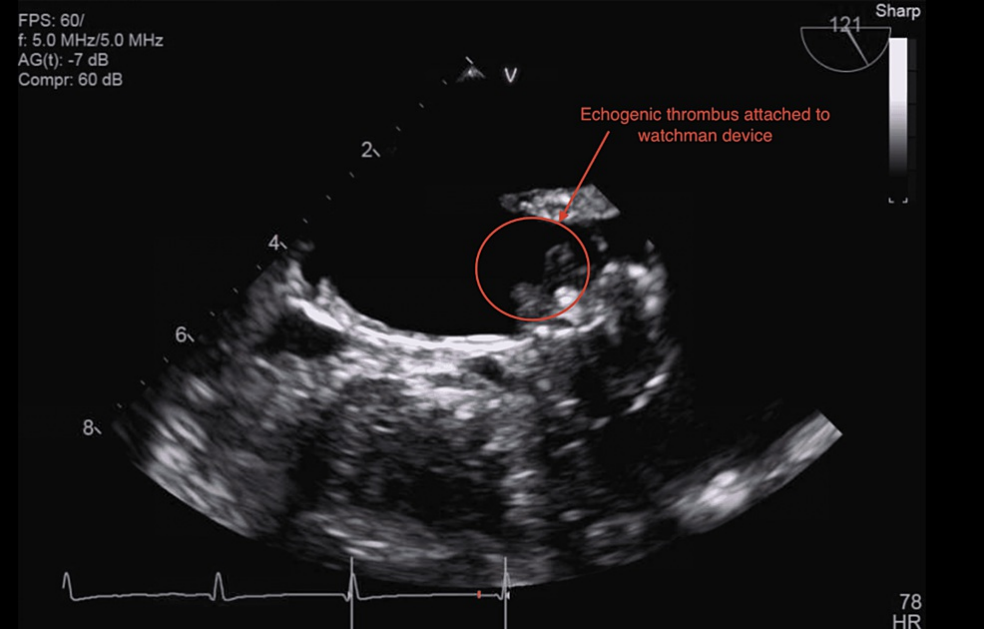
Thrombus on the WD (circle). WD, watchman device

**Figure 2:**
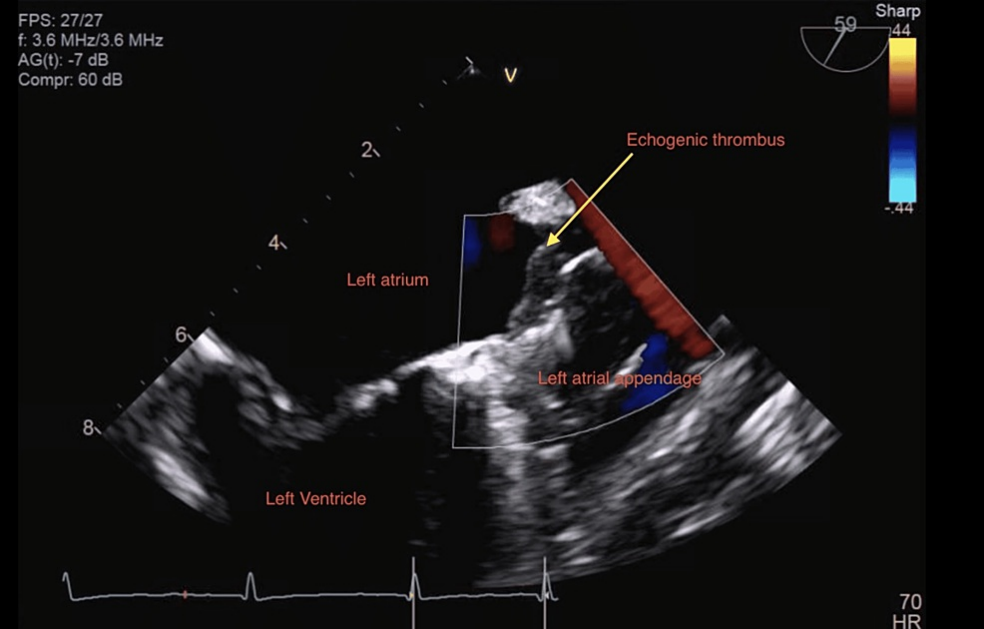
Echogenic thrombus on the WD (arrow). WD, watchman device

The patient is an 84-year-old female with a history of bleeding peptic ulcer disease and traumatic subarachnoid hemorrhage, hypertension, hyperlipidemia, and diabetes mellitus type 2 initially admitted to the hospital with a chief complaint of expressive aphasia. After 30 min the patient's speech became normal and her initial National Institute of Health stroke scale was 0 on arrival at the emergency department. Tissue plasminogen activator (TPA) was not given due to the complete resolution of the patient's symptoms. She underwent a CT head which did not show any intracranial bleeding. She then underwent an MRI of the brain which showed chronic ischemia and an old lacunar infarct. Neurology was consulted. The patient was not on aspirin at home due to a previous bleeding peptic ulcer. The patient was started on aspirin this time for TIA, on a rhythm strip patient was found to have atrial fibrillation; on further questioning, the patient stated she was not on any anticoagulation for the last 16 months after LAAC was done with 27 mm "watchman device" (WD). The patient underwent TEE 45 days after LAAC without evidence of any peri-device leak or thrombus. She was on apixaban for initial 45 days after the procedure, and she was not on any antiplatelet. However, she never got follow up (FU) TEE at six months due to other health issues and failed to FU. Interventional cardiology was consulted, and the patient underwent a transthoracic echocardiogram (TTE) which showed her ejection fraction was 45%-50%. Subsequently, the patient underwent TEE which showed a large 1.5 cm x 0.8 cm fixed echogenic thrombus attached to the WD (Figures 1-2).

The watchman closure device completely occludes the left atrial appendage with an echogenic thrombus attached to the WD (Video 1). Carotid ultrasound showed mild bilateral internal carotid artery stenosis. The patient has been started on aspirin. After the TEE, the patient was also started on heparin infusion for further stroke prevention, eventually transitioning to factor Xa inhibitor apixaban. Heparin was chosen due to its short half-life and can be reversed easily if the patient bleeds from a previous peptic ulcer. The patient was kept in the hospital for 48 h for close monitoring. The patient tolerated anticoagulation well with no further bleeding episodes and stable hemoglobin. The patient was discharged home, and the patient came back to FU after one month with no further stroke-like symptoms. As her symptoms resolved she declined to have a repeat TEE done to evaluate the status of the thrombus. 

**Video 1:** TEE showing thrombus attached to the WD. WD, watchman device; TEE, transesophageal echocardiogram

## Discussion

Patients with contraindication to take long-term anticoagulation LAAC surgery is an option to prevent thromboembolism [1]. One of the common causes of embolic stroke is atrial fibrillation. The WD is the only percutaneous left atrial closure device that has shown efficacy and safety for the prevention of stroke and systemic embolization approved by the US Food and Drug Administration (FDA). The WD was evaluated in two randomized trials PROTECT AF (watchman left atrial appendage system for embolic protection in patients with atrial fibrillation) and PREVAIL trial (evaluation of the watchman LAA closure device in patients with atrial fibrillation versus long term warfarin therapy) in patients with nonvalvular AF eligible for oral anticoagulation [2-3]. Based upon the results of PROTECT AF and PREVAIL trials, FDA approved the WD in March 2015. After FDA approval long-term complication of the device has been evaluated and more data came out regarding DRT and the potential risk for thromboembolic complications. A recent multiple study looked at the data for DRT although it is rare, but DRT was found to have caused more systemic thromboembolism [4-5]. Of 1739 patients who received an implant, DRT was seen in 65 patients (3.74%) [4]. The rates of systemic thromboembolism with and without DRT were 7.46 and 1.78 per 100 patient-years and ischemic stroke rates were 6.28 and 1.65 per 100 patient-years [4]. The most common predictors of DRT were: a history of transient ischemic attack (TIA) or stroke, permanent AF, vascular disease, left atrial appendage diameter, and left ventricular ejection fraction. DRT and systemic thromboembolism both occurred in 17 of 65 patients (26.2%). Of the 19 systemic thromboembolism events in these patients with DRT, 9 of 19 (47.4%) and 12 of 19 (63.2%) occurred within one and six months of DRT detection. According to the study, DRT was associated with a higher risk of systemic embolism [4]. Other study DRT was detected after a median of 93 days, with 82.1% (128/156) of cases being detected within six months after LAAC [5]. The remaining 17.9% were detected during FU after a median of 304 days. In both of the studies most cases of DRT with systemic thromboembolism were identified within the first 365 days of the watchman procedure. Our patient’s TIA from DRT was identified after almost 480 days. The patient was lucky to have only TIA with a short period of expressive aphasia with complete resolution of symptoms. The patient was initially treated with heparin infusion eventually transitioning to factor Xa inhibitor apixaban. Our patient underwent both TTE and TEE with TTE failing to show any thrombus on the WD, however, TEE revealed a 1.5 cm x 0.8 cm fixed echogenic thrombus attached to the WD. Prompt identification and treatment with systemic anticoagulation potentially prevented larger strokes which could cause significant disability or even death. The optimum antithrombotic regimen for DRT is unknown. Options are a combination of direct oral anticoagulation or warfarin or low molecular weight heparin therapy and aspirin [6].

## Conclusions

It is important to consider WD-related thrombosis as a cause of systemic thromboembolism even after 12 months of the procedure, especially in patients who are not on anticoagulation. It also raised concern about discontinuing systemic anticoagulation in patients with persistent AF who underwent LAAC. There was a patient's need to be educated about common symptoms of stroke and the risk of cerebrovascular accidents even after the first 365 days of the surgery. Our patient was successfully treated with systemic anticoagulation with apixaban and aspirin with 30-day prevention of recurrent stroke.
